# Artichoke Biorefinery: From Food to Advanced Technological Applications

**DOI:** 10.3390/foods10010112

**Published:** 2021-01-07

**Authors:** Matteo Francavilla, Mauro Marone, Paolo Marasco, Francesco Contillo, Massimo Monteleone

**Affiliations:** STAR Research Group, Department of Agriculture, Food, Natural Resources and Engineering (DAFNE), University of Foggia, Via Napoli 25, 71122 Foggia, Italy; mauro.marone@unifg.it (M.M.); paolo.marasco@unifg.it (P.M.); francesco.contillo@unifg.it (F.C.); massimo.monteleone@unifg.it (M.M.)

**Keywords:** globe artichoke residues, biorefinery, microwave assisted extraction, phenols, inulin

## Abstract

A sequential extraction process has been designed for valorizing globe artichoke plant residues and waste (heads, leaves, stalks, and roots left in the field) by means of green extraction techniques according to a biorefinery approach. We investigated two cascading extractions based on microwave-assisted extractions (MAE) and green solvents (water and ethanol) that have been optimized for varying temperature, solvent and extraction time. In the first step, phenols were extracted with yields that ranged between 6.94 mg g^−1^ dw (in leaves) and 3.28 mg g^−1^ dw (in roots), and a phenols productivity of 175.74 kg Ha^−1^. In the second step, inulin was extracted with impressive yields (42% dw), higher than other conventional inulin sources, corresponding to an inulin productivity of 4883.58 kg Ha^−1^. The remaining residues were found to be valuable feedstocks both for bioenergy production and green manure (back to the field), closing the loop according to the Circular Economy paradigm.

## 1. Introduction

The consumption of raw materials such as biomass, fossil fuels, metals, and minerals is expected to double over the next 40 years on a global scale [1]. Likewise, the waste generated each year is estimated to increase by 70% by 2050 [2]. With that in mind, the European Commission has recently enforced the Circular Economy Action Plan [3], one of the main components of the overall European Green Deal, the European new agenda for sustainable growth [4]. The Green Deal is implementing an EU coordinated strategy to become a climate-neutral, resource-efficient, and competitive economy. Achieving climate neutrality by 2050 will require a decisive effort in supporting the scaling up of circular economy from front-runners to the mainstream economic players; in this way, a decoupling of economic growth from resource use will be encouraged, and the long-term competitiveness of the EU will also be fostered.

Small and medium-sized enterprises are the leading actors of this ecological shifting by adopting advanced and innovative “green” technologies and putting into practice the appropriate know-how, got from Academia, for achieving the replacement of hazardous substances from the production processes. This approach can be applied in almost all manufacturing sectors. The agro-industrial field is of great interest because of the huge amount of residues and by-products that are generated. They could be converted into high value compounds and products by means biorefineries based on environmentally friendly processes [5,6]. On the other hand, the effective deployment of integrated biorefinery plants requires the setting up of reliable processing units combined with advanced eco-friendly and economically profitable value chains [7]. Increasing use of disposable, underused, and residual biomass could increasingly supply the feedstock requirements as expressed by forthcoming developments of new integrated biorefinery systems.

In this context, globe artichoke (*Cynara cardunculus* L. subsp. *scolymus* (L.) Hayek) represents a very intriguing feedstock for the Mediterranean Area. It is a perennial plant belonging to the Asteraceae family and originating from the Mediterranean region [8]. It is traditionally cultivated as a polyannual crop through vegetative propagation. Nevertheless, the length of the crop cycle negatively influences yields and the quality of the heads. This led artichoke growers to take an interest in the development of new seed-propagated cultivars for annual crops [9,10,11]. The world production of artichoke accounts for about 1678 Ktons, and Italy is the largest producer with 390 Ktons of artichokes in the world, followed by Egypt (324 Ktons), Spain (208 Ktons), and Perù (155 Ktons) [12]. Heads (flowers) and stems just below them constitute the edible part of the plant. They are characterized by a high content of bioactive compounds, including phenols, inulin, fibers, and minerals that make them really attractive for market [13,14,15]. The industrial artichoke processing generates a huge amount of waste biomass (80–85%) unsuitable for human consumption that is composed of bracts and stems cut during the harvesting process [14,16]. However, according to Gominho et al. [8], it represents only a small part (15–30% dw) of the entire biomass, depending on genotype, climate, soil, and culture conditions. The remaining part (70–85% dw) is composed by residual leaves, stalks and roots that remain available in the field when cultivated as an annual crop and are usually disposed of as solid waste or left in the field without any further valorization. 

Interestingly, the phenolic composition of these residues have been proved to be similar to the edible parts of the plant [17,18,19]. Moreover, according to Zuorro et al. [16], the phenolic content is higher than other phenols source such as carrot peels, grape pomace, and spent coffee grounds. In addition, an interesting amount of inulin in artichoke roots (6–21% dw) was recently reported by Castellino et al. [9].

Phenols and inulin from residual biomass of artichoke are excellent candidates for the biorefinery process having many therapeutic properties and biotechnological applications that could contribute in increasing the economic value of residual biomass. The artichoke phenols have been identified since ancient times for their beneficial effects and therapeutic actions including the promotion of blood circulation, mobilization of energy reserves, induction of choleresis, inhibition of cholesterol biosynthesis and low-density lipoprotein (LDL) cholesterol oxidation. A significant antibacterial, antifungal, and antioxidant, as well as strong hepatoprotective effects, are also recognised [15,20,21]. Inulin is a natural storage polysaccharide widely distributed in plants. It is a water-soluble fiber that consists of a mixture of oligo- and polysaccharides of β(2→ 1) linked D-fructose units with a terminal glucose residue, which are classified as fructans [22]. Inulin-type fructans are considered as prebiotic compounds, i.e., it is indigestible by humans, but it stimulates the growth and activity of specific microorganisms, including *Lactobacilli* and *Bifidobacteria* in the colon [23,24,25].

Following a general trend towards the recycling biomass, including food waste and agro-industrial residues, several research studies have been performed in order to find new pathways for valorizing the residues of artichoke [26]. Some examples include the extraction of phenolic compounds [16,27,28], the production of biofuels [14,29], the recovery of enzyme peroxidase for wastewater treatment [30], and the extraction and purification of high-molecular-weight inulin [9,31]. In the light of our knowledge, little attention has been paid in sequential and eco-friendly processes that could convert residual artichoke biomass into a plethora of high-value compounds (phenols, inulin, etc.), bioenergy and agricultural application at the same time. Nevertheless, no studies have been performed aiming at designing an integrated biorefinery process for the valorization of different parts of globe artichoke plant residues (heads, leaves, stalks, and roots) when cultivated as an annual crop.

Therefore, this work aimed to investigate a sequential microwave extraction process of phenols and inulin from artichoke crop residues, inspired to green chemistry principles, to convert waste into valuable compounds with a biorefinery approach. Moreover, two different valorization pathways of extracted residual biomass as bioenergy feedstocks and green manure for agriculture application were evaluated.

## 2. Materials and Methods

### 2.1. Sampling and Biomass Preparation

Artichoke plants (*C. cardunculus* L. subsp. *scolymus* (L.) Hayek), Madrigal^®^ (Nunhems SAS, Beaucouze, France) seed propagated cultivar (hybrid variety), were collected at the end of the productive stage of heads (June 2019, after harvesting for market) from a 4 Ha cultivated field (FIMAGRI Farm) located at Candela in Southern Italy (latitude 41°08′ N, longitude 15°31′ E altitude 499 m above sea level). We randomly selected four sampling squares (3.5 m × 3.5 m) in the field. The number of plants was counted in each sampling unit. Then, three entire plants along the diagonal of each square were collected. Sampled plants (12) were immediately brought to the laboratory where the 4 main components were hand separated: Heads (residual), leaves, stalks and roots. The wet weight was measured for each component. Samples were dried in a ventilated oven at 60 °C according to National Renewable Energy Laboratory (NREL) protocol [32]. Dried samples were ground in a cutting mill (Pulverisette 15, Fritsch, Idar-Oberstein, Germany) to pass through a 1 mm sieve. The mixed portions of dried tissue were used for chemical characterization and extractions.

### 2.2. Chemical Characterisation of Biomass

Proximate analysis (moisture, ash, volatile solids, fixed carbon) of raw biomass (heads, leaves stalks and roots) and extracted biomass, after the sequential extraction of phenols and inulin, was performed using a thermogravimetric analysis system (TGA 701, LECO, St. Joseph, MI, USA), following ASTM D7582 method.

Ultimate analysis (C, H, N, S, O) was performed using a CHNS628 elemental analyzer (LECO, St. Joseph, MI, USA) and following the method LECO-ASTM-D 5291. The oxygen content was calculated by difference, including the ash content.

Protein content was calculated by multiplying elemental N concentration by a factor of 6.25, according to standard method AOAC-2016.

The high heating value (HHV) was experimentally determined in the laboratory with an adiabatic bomb calorimeter AC-500 (LECO, St. Joseph, MI, USA) following the standard method CEN/TS 14918:2005.

Elemental analysis (micro- and macro-elements) was performed by digesting 0.25 g dw of the sample in 20 mL of HNO_3_ in a closed vessel of microwave digester (CEM-Mars6) for 20 min at 220 °C. The metals in the solution were analyzed by inductively coupling plasma optical emission spectroscopy (ICP-OES Agilent 720, Agilent Technologies, Santa Clara, CA, USA), calibrated with external standard (TraceCERT^®^, Sigma–Aldrich, St. Louis, MO, USA).

Structural-carbohydrates from cellulose and hemicelluloses together with ‘‘Klason lignin” were measured using a strong acid hydrolysis, according to National Renewable Energy Laboratory (NREL) method [33]. Monosaccharides (i.e., glucose, xylose, arabinose, fructose, galactose), uronic sugars (glucuronic and galacturonic) and dehydration products (levulinic acid, hydroxyl methyl furfural, and furfural) were analyzed by HPLC (Agilent 1260, Agilent Technologies, Santa Clara, CA, USA) coupled to a refractive index detector (RID). The analysis was carried out with a Hi-Plex H column at 60 °C. The eluent was ultrapure water (MilliQ^®^, Merk Millipore, Burlington, MA, USA) 5 mM H_2_SO_4_ under a flow rate of 0.7 mL min^−1^. The system was calibrated with pure chemicals from Sigma–Aldrich (St. Louis, MO, USA). Thereafter, cellulose and hemicelluloses contents were estimated as follows:

Cellulose (% dw) = Glucose (% dw)/1.11

Hemicelluloses (% dw) = [Xylose (% dw) + Arabinose (% dw)]/1.13

where 1.11 is the conversion factor for glucose-based polymers (glucose) to monomers and 1.13 is the conversion factor for xylose-based polymers (arabinose and xylose) to monomers.

Total Carbohydrates were quantified as the sum of monomeric carbohydrates, uronic sugars, and dehydration products (levulinic acid, hydroxymethylfurfural, and furfural) converted into corresponding hexose and pentose sugars amount as follows:

Hexose = Levulinic Acid (% dw) × 1.55

Hexose = Hydroxymethyl furfural (% dw) × 1.43

Pentose = Furfural (% dw) × 1.56

where 1.55 is the hexose–LA molecular weight ratio, 1.43 is the hexose–HMF molecular weight ratio and 1.56 is the pentose–Furfural molecular weight ratio.

### 2.3. Microwave-Assisted Extraction (MAE) of Phenols

Microwave extraction was carried out using a microwave reaction system MARS-6 (CEM srl, Cologno Al Serio, Italy) consisted of 12 closed extraction vessels equipped with an infrared temperature sensor. Direct measurement of pressure and temperature (by means optic fiber probe) was performed in the reference vessel. The magnetic stirring was set at 300 rpm. There were two green solvents, water, and ethanol at four different concentrations in water (0, 25, 50, and 75% *v/v*), used for the extraction. The sample weight to solvent volume ratio was maintained constant at 1:10 (*w/v* ratio) for all extractive experiments. In a typical extraction process, 1 g dw of biomass was extracted with 10 mL of solvent. We tested three temperature levels (50, 75 and 100 °C) with three different extraction times (5, 10, and 20 min). The microwave frequency used for the extraction was 2450 MHz. At the end of the extraction, the vessels were cooled down to 25 °C using compressed air. Then, the mixture was centrifuged at 4100 rpm for 10 min, and the liquid phase was filtered (0.20 µm). The extracts were then flushed with nitrogen gas and stored in the dark at −40 °C until HPLC analysis of phenolic profile. The wet solid residue (Phenols Extracted Residue—PER) was weighed and stored for sequential inulin extraction. A portion was dried in an oven at 60 °C, weighed and stored for further chemical characterizations.

### 2.4. Conventional Extraction (CE) of Phenols

With the aim to evaluate the extraction efficiency of MAE, the phenols extraction was performed following the conventional extraction (CE) method shaking the biomass with solvents reported above for MAE (1:10, *w/v* ratio) for 20 min, 8, 24, and 48 h at room temperature and using an orbital shaker (Stuart, Cole-Parmer srl, Cernusco sul Naviglio, Italy) set at 70 rpm. The following work sequence was the same as described above for MAE.

### 2.5. Microwave-Assisted Extraction of Inulin

Microwave-assisted extractions (MAE) of inulin from raw and residual biomass (PER) were performed in the same microwave reaction system (MARS-6, CEM srl, Cologno Al Serio, Italy) used for phenol extraction. Sample (0.5 g dw) was transferred to an extraction vessel containing 50 mL of water (MilliQ^®^, Merk Millipore, Burlington, MA, USA). The operational parameters employed in the MAE apparatus were the following: magnetron power 100%, ramp temperature and extraction time of 5 min, respectively. During operations, both temperature and pressure were monitored. Then, three temperature levels were tested for extractions: 60, 80, and 100 °C. After the extraction, the vessels were cooled down to room temperature, and the mixture was centrifuged at 4100 rpm for 10 min. The liquid phase was filtered (0.45 µm) and analyzed by HPLC for inulin quantification. The extracted inulin was precipitated with EtOH, dried and then analyzed by FT-IR ATR (Attenuated Total Reflectance). The solid residue (Inulin Extracted Residue—IER) was dried in an oven at 60 °C, weight and stored for further chemical characterizations. The inulin extraction efficiency of MAE compared to CE was evaluated.

### 2.6. Conventional Extraction of Inulin

The conventional extraction of inulin was performed by means hot water diffusion procedure at an average temperature of 85 °C for one hour with a continuous stirring as reported by Zutela and Sambucetti [34]. The sample (1 g dw) was transferred into 200 mL Pyrex beaker, added with 100 mL of hot water at pH 6–8, and kept at 85 °C with continuous magnetic stirring for 1 h. The mixture was then cooled down to room temperature, and the volume was made up to 100 mL. The solution was filtered (0.45-µm) and characterized by HPLC analysis.

### 2.7. HPLC Analysis of Phenolic Profile

The extracted phenols were analyzed by means high pressure liquid chromatography (HPLC, 1260 Infinity, Agilent Technologies, Santa Clara, CA, USA) coupled with a Diode Array Detector (DAD). A reversed-phase Zorbax Stable Bond SB-C18 column (250 × 4.6 mm i.d., particle size 5 µm, Agilent Technologies, Santa Clara, CA, USA) with a Zorbax precolumn guard cartridge (10 mm × 4 mm i.d. 5 µm) was used for chromatographic separation at room temperature (25 °C). The injection volume was set at 20 µL.

A gradient binary elution was performed using solvent A (0.1% formic acid in water) and solvent B (0.1% formic acid in acetonitrile/methanol, 60:40 *v*/*v*) at a constant flow of 0.8 mL min^−1^. The gradient program reported by Rouphael et al. [13] was used: 20–30% B (6 min), 30–40% B (10 min), 40–50% B (8 min), 50–90% B (8 min), 90–90% B (3 min), 90–20% B (3 min).

HPLC was calibrated using commercial standards provided by Sigma–Aldrich (St. Louis, Missouri, USA): chlorogenic acid (≥96% purity), caffeic acid (≥97% purity), 1,3-dicaffeoylquinic acid (≥95% purity), ferulic acid (≥95% purity), 1,5-dicaffeoylquinic acid (≥95% purity), 1-caffeoylquinic acid (97% purity), apigenin (95% purity), luteolin (≥95% purity), apigenin-7-O-glucoside (≥95% purity), luteolin 7-O-glucoside (≥95% purity).

Caffeoylquinic derivatives were quantified at 330 nm, while apigenin and luteolin derivatives were quantified at 330 nm and 350 nm, respectively. Phenolic compounds were identified by comparison with commercial standards and available data in the literature [13,35]. All data are reported as mg g^−1^ of dry matter (dw). Calibration curves and calculation of limit of detection (LOD) and limit of quantification (LOQ) values are reported in Table S1 (Supplementary Materials).

### 2.8. HPLC Analysis of Inulin

Inulin was quantified by HPLC analysis following the analytical method reported by Zutela and Sambucetti [34]. The chromatographic equipment consisted of an Agilent 1260 HPLC (Agilent Technologies, Santa Clara, CA, USA) equipped with a refractive index detector and an Aminex HPX-87C (Bio-Rad Laboratories S.r.l., Segrate, Italy) anion exchange column. The mobile phase was ultrapure water (MilliQ^®^, Merk Millipore, Burlington, MA, USA) at 85 °C at a flux rate of 0.6 mL min^−1^ and an injection volume of 20 µL. Pure inulin from chicory (Sigma–Aldrich, St. Louis, MO, USA) was used as an external standard for calibration. A calibration range between 0.5 and 5 mg mL^−1^ was set for inulin quantification.

### 2.9. FT-IR ATR Analysis of Inulin

Standards and mixtures were analyzed by Fourier transform infrared attenuated total reflectance (FT-IR ATR) spectroscopy by Perkin Elmer Spectrum TWO FT-IR spectrometer equipment (Waltham, MA, USA) operating at 4 cm^−1^ resolution with 64 scans per test. Spectra were collected in the transmittance mode from 4000 to 400 cm^−1^.

### 2.10. Statistical Analysis

All the experiments were repeated three times. Unless otherwise stated, all data were expressed as mean ± standard deviation (SD). The means of all the parameters were examined for significance by analysis of variance (ANOVA) using the software JMP version 9 (SAS Institute Inc., Cary, NC, USA). When F values showed significance, individual means were compared using Tukey’s honest significant difference (HSD). Significant differences were considered when *p* < 0.05.

## 3. Results and Discussion

### 3.1. Waste Biomass Available in the Field

Globe artichoke (*C. cardunculus* var. *scolymus* L.), cultivar Madrigal^®^ (Nunhems SAS, Beaucouze, France), was cultivated as an annual crop instead of multi-years cycles. The availability of artichoke crop residues in the field is reported in Table 1. After heads harvesting, the residual biomass left in the field was about 33 tons dry weight (dw) per hectare. Leaves, stalks, and roots represented roughly one-third of total biomass (10.54 t dw Ha^−1^, 10.68 t dw Ha^−1^ and 11.16 t dw Ha^−1^, respectively). This data are higher than those recently reported by Pesce et al. [36] who found for three different cultivars (Spinoso sardo, Violetto di Sicilia, and Apollo) residual biomass in the field (including the aboveground biomass only) that ranged between 5.1 t dw Ha^−1^ and 13.8 t dw Ha^−1^. Furthermore, the amount of Madrigal residual crop is very high if compared with other annual residual crops including wheat, barley, rye, oat, maise, rice, chickpea, lentil, cotton, sunflower, soybean, and tobacco. Almost all of these crops produce on average less than 5 t dw Ha^−1^ of post-harvest residues [37]. Interestingly, the amount of Madrigal artichoke crop residues is very close to the biomass produced by cultivated cardoon (both belong to the same species) that can produce up to ~36 t dw Ha^−1^ [38].

Considering that total cultivated area in Italy for producing globe artichoke amounted to 40,175 Ha in 2018, it is clear how intriguing is the interest in developing a convenient and efficient process to valorize such a resource according to Circular Economy paradigm.

### 3.2. Chemical Characterisation of Raw Biomass

The residual biomass left in the field has been fractionated in four main components: heads (residual heads after harvesting), leaves, stalks and roots. They were characterized in terms of proximate and ultimate analysis (Table 1).

The aboveground parts of plants showed a higher moisture content (82–87% w) than roots (66% w). Leaves and roots showed a similar content of ash (14.80 and 17.58% dw, respectively), whereas a lower content was found in heads and stalks (7.63 and 6.40% dw) as reported by Gominho et al. [8] for *C. cardunculus* L.: The carbon content (C) was almost homogeneous in aboveground parts (about 40%) and slightly lower in roots (36.25%). The amount of hydrogen (H) was found at the same level in the four plant components (about 6.3% dw). Nitrogen was more concentrated in heads and leaves (2.49% dw and 2.77% dw, respectively) than in stalks and roots (0.87% dw and 0.74% dw, respectively). Proteins followed the same trend of nitrogen with higher content in heads and leaves (15.58% dw and 17.32% dw, respectively) than in stalks and roots (5.44% dw and 4.61% dw, respectively), according to results reported by Zuorro et al. [14] and Rouphael et al. [13].

The highest amount of cellulose, hemicellulose and lignin was found in stalks (24.15% dw, 10.86% dw and 15.62% dw, respectively). Lower concentrations were found in the other investigated plant components. Different values were reported by Pesce et al. [36], who found in Spinoso sardo cultivar 23.62% dw of cellulose, 17.70% dw of hemicellulose and 10.54% dw of Klason lignin. However, biomass composition can be affected by environmental conditions, seasonal trends and cultivation practices.

Total carbohydrates that include structural and not structural carbohydrates ranged between 65% dw (in leaves) and 56% dw (in heads).

### 3.3. Sequential Process

**Figure 1:**
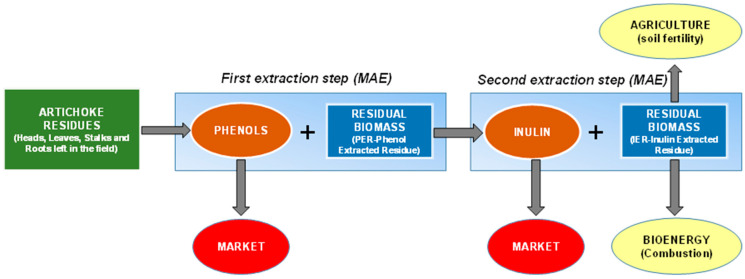
The sequential process designed for valorizing artichoke plant residues left in the field with a biorefinery approach.

The sequential process designed for valorizing different globe artichoke plant residues (heads, leaves, stalks, and roots left in the field) with a biorefinery approach is shown in Figure 1. The two cascading eco-friendly extraction processes based on microwave-assisted extraction (MAE) and green solvents (water and ethanol) have been optimized. In the first step, phenols are extracted from raw biomass generating a marketable product (phenols extract) and a by-product (PER-Phenol extracted residue). In the second extraction process, PERs are extracted, producing a further marketable product (inulin) and a by-product (Inulin extracted residue—IER)). IERs have been characterized for their possible valorization as bioenergy feedstock and for agriculture application (green manure).

#### 3.3.1. First Step: Microwave-Assisted Extraction (MAE) of Phenols

**Figure 2:**
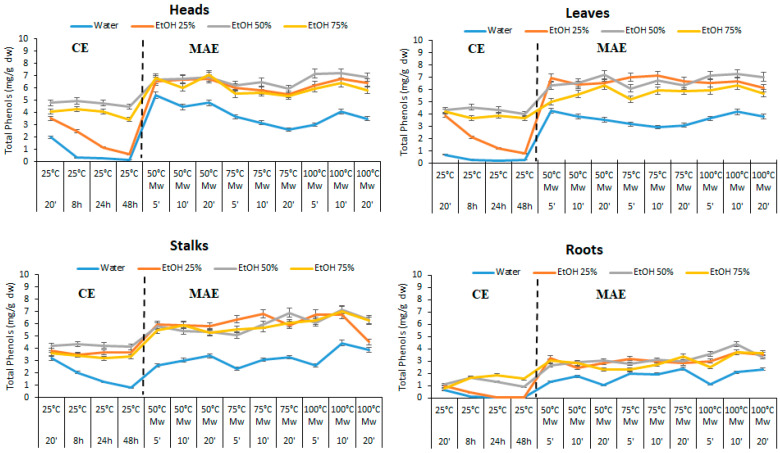
Total phenols extraction yields (mg g^−1^ dw) from residual artichoke biomass (heads, leaves, stalks and roots) achieved by conventional extraction (CE) and microwave assisted extraction (MAE) at different experimental conditions (solvent, time, and temperature).

The yields of total phenols (TP) extracted by MAE and CE from residual artichoke biomass are depicted in Figure 2 (Table S2, Supplementary Materials). The results showed a high variability depending on method and extraction conditions. Microwave extraction (MAE) was more efficient than conventional extraction (CE) for all experiments regardless of time and solvent. MAE’s yields in some experiments were 10 times higher than conventional extraction ones (6.9 mg g^−1^ dw by MAE vs. 0.67 mg g^−1^ dw by CE with EtOH 25%).

Solvent effect. The solvent selection was a key factor both for MAE and CE. EtOH 25% and EtOH 50% in water gave the best yields in MAE while EtOH 75% gave phenols yields that were slightly lower. Pure water was the worst solvent. Pradal et al. [39] reported a similar effect on phenol extraction from chicory waste. They found that EtOH concentration higher than 60% *v/v* generated a decrease in extraction yield and phenols recovery. Pure EtOH was found to be ineffective. On the other hand, using a lower amount of EtOH can make the entire process more sustainable in an economic and environmental point of view. Actually, in a recent Life Cycle Assessment (LCA) analysis, Santiango et al. [40] found that the conventional extraction of phenols (flavonols), based on the use of ethanol (70% *v/v* in water) as an extractive agent, had significant environmental burdens due to its background production processes. Therefore, the reduction of EtOH percentage and non-conventional extraction techniques (including MAE) was suggested for reducing the environmental impact.

In conventional extraction (CE) the highest yields were found using EtOH 50% and EtOH 75% while lower yields were achieved by using EtOH 25%. Pure water was confirmed as the worst solvent in CE as well. This was mainly related to the higher polarity of water (µ = 1.85 D) than ethanol (µ = 1.69 D), making phenols less soluble in pure water. In the MAE process, the extraction yield depends on a balanced effect between the solubility of phenols in the solvent, and the energy transfer from the solvent to the matrix, depending on dielectric constant (ε) of solvent and temperature. Water and ethanol are good microwave absorbers (ε = 87.4 for water and ε = 24.8 for ethanol at 25 °C), resulting in the right choice for MAE [41].

In many studies, a solid/solvent ratio from 1:10 (g/mL) to 1:100 (g/mL) was reported to be the best condition both for MAE and CE [27,41,42]. A biomass–solvent ratio 1:10 (g dw/mL) was selected for all the experiments in the present work. The solvent amount was enough to immerse the biomass completely. Moreover, this approach generated, between others, two main beneficial effects: a huge solvent saving and high concentrated phenols extracts (0.7 mg mL^−1^).

Time effect. The effect of time and temperature were also investigated. Over-exposure to micro wave radiation for longer times (>30 min) can determine a decrease in the extract yield and phenol recovery due to the degradation of bioactive organic compounds present in the biomass [43]. In the present study, no significant differences were found between 5 and 10 min as extraction time. Longer time (20 min), when associated with high temperature (100 °C), generated a slight yield decrease (Figure 2).

Temperature effect. Concerning temperature, it is demonstrated that a prolonged exposure to high temperatures can generate thermal degradation of phenols [44]. Moreover, the higher and longer the temperature and extraction time, the higher the energy consumption and the overall process cost. Therefore it is strategic to find the right compromise between all factors and parameters that can influence the extraction process.

Selection of best extraction condition. With all that in mind, the best conditions we selected for MAE phenols extraction were: solvent EtOH 25% (*v/v* in water); time 5 min, temperature 50 °C. The total phenols yields obtained from heads, leaves, stalks, and roots with the above-described extraction conditions are shown in Figure S1 (Supplementary Materials). Data are reported as total phenols (TP), namely the sum of identified phenolic compounds by HPLC. Leaves were found to have the highest TP content (6.94 mg g^−1^ dw) between the aboveground parts of the artichoke plant. Heads and stalks showed a TP content of 6.52 mg g^−1^ dw and 5.93 mg g^−1^ dw, respectively. The lowest TP concentration was found in roots (3.28 mg g^−1^ dw).

Phenols composition of extracts at optimized MAE conditions. Within the caffeoyl derivatives, chlorogenic acid (5-O-caffeoylquinic acid) was the most abundant component in leaves and stalks (3.89 mg g^−1^ dw and 2.79 mg g^−1^ dw, respectively), while the 1,5-O-dicaffeoylquinic acid was the most abundant in heads (2.78 mg g^−1^ dw) followed by stalks (2.45 mg g^−1^ dw) (Table 2). Mena-Garcìa et al. [27] reported a concentration of chlorogenic acid of 1.56 mg g^−1^ dw and 2.39 mg g^−1^ dw in heads and stalks, respectively. In that study, the extraction was performed by a microwave assisted process (MAE) at 97 °C, 3 min and ethanol:water (50:50, *v/v*). Concentrations of chlorogenic acid in leaves ranging between 0.6 to 9 mg g^−1^ dw were reported by Gouveia and Castilho [45] and Mulinacci et al. [46] for Portuguese and Italian artichoke leaves, respectively. Other main caffeoylquinic derivatives identified in our residual artichoke biomass were cynarin (1,3-O-diffeoylquinic acid) and 3,4-O-dicaffeoylquinic acid. Interestingly, the highest cynarin content was found in roots (0.15 mg^−1^ dw).

Besides caffeoylquinic derivatives, flavones luteolin and apigenin were identified and quantified. Luteolin 7-O-glucoside, luteolin 7-O-glucoronide and luteolin 7-O-rutinoside were found only in stalks extracts with concentrations between 0.60 mg g^−1^ dw and 0.26 mg g^−1^ dw. Apigenins were more abundant in heads with concentrations that ranged between 0.25 mg g^−1^ dw and 0.008 mg g^−1^ dw. According to Lattanzio et al. [15], these compounds are minor constituents of total phenolic content (about 10% or less) from a quantitative point of view.

#### 3.3.2. Second Step: MAE Inulin Extraction

With the aim to set up a cascading process for artichoke biomass valorization, wet solid residues (PERs) resulting from optimized MAE phenols extraction were tested for inulin extraction by MAE using water as a solvent. Firstly, the inulin content in raw biomass was analyzed using the conventional extraction method. Secondly, the MAE method was optimized for both raw and extracted (PER) biomass in order to evaluate how the first extraction process (phenols) could negatively affect the inulin yields.

**Figure 3:**
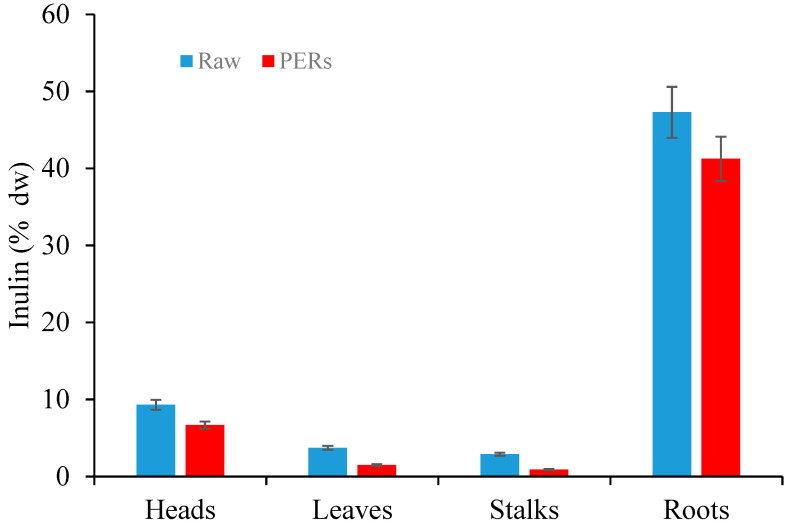
Inulin yields achieved by MAE (5 min, 80 °C, 1:10 *w/v* ratio) from artichoke biomass (Heads, Leaves, Stalks, and Roots) before (Raw) and after phenols extraction (Phenols Extracted Residues—PERs).

Figure 3 shows the inulin yields achieved from artichoke biomass before (Raw) and after (PER) phenols extraction. Inulin was extracted by MAE using water as solvent at 80 °C for 5 min and biomass to solvent ratio of 1:10 *w/v*. Higher temperature (up to 100°C) and lower temperature (up to 60 °C) resulted in a decrease in inulin yield (data not reported). Moreover, no differences were found between inulin yields achieved by the optimized MAE method (5 min, 80 °C, 1:10 *w/v* ratio) and CE method (1 h, 85 °C, 1:10 *w/v* ratio), resulting in high extraction efficiency (>98%) for MAE.

Inulin content in roots (47% dw) was impressively higher than those reported by Castellino et al. [9] for the same artichoke cultivar Madrigal^®^ (Nunhems SAS, Beaucouze, France) (12% dw) extracted by ultrasounds assisted extraction in the water at the same temperature. However, besides extraction method, pedoclimatic variables and harvesting time can show significant effects on extraction yields. Interestingly, inulin content found in our artichoke roots was higher than other conventional inulin sources recently reviewed by Singh et al. [23]: Agave (7–10% ww), Camas (12–22% ww), Chicory (15–20% ww), Dahlia (15–20% ww), and Jerusalem artichoke (12–19% ww).

Inulin content detected in heads was 9.30% dw according to data reported by Lattanzio et al. [15]. Leaves and stalks showed a lower inulin content that ranged between 3.7% dw and 2.9% dw, respectively (Figure 3). In previous work, Ruiz–Aceituno et al. [47] investigated the extraction of inulin from artichoke’s external bracts (var. Blanca de Tudela) using MAE. Under their best MAE conditions (120 °C, 3 min and a solid:solvent ratio of 1:33 g/mL), they achieved a lower inulin yield (1.14% dw) compared to our findings.

Comparing inulin yields achieved from raw biomass and PERs, a decrease in inulin content was found in each plant component highlighting how the phenols extraction can negatively affect inulin yield (Figure 3). The inulin content in PERs of roots and heads was 41% dw and 7% dw, respectively. However, inulin yield from PERs of roots remained impressively higher than other conventional sources.

FT-IR ATR analysis. FT-IR ATR analysis of inulin extracted by MAE from raw and extracted roots was performed and compared with commercial inulin from chicory (Sigma–Aldrich, St. Louis, MO, USA) as reference (Figure S2, Supplementary Materials). The FT-IR region spectrum between 1500 to 1200 cm^−1^ displays absorption bands belonging to single bond atoms [48]. However, signals between 1200 to 900 cm^−1^, which are generally recognised as the fingerprint, are the most informative used in FT-IR analysis of carbohydrates [48,49].

According to data recently reported by Vàsquez–Vuelvas et al. [50], inulin spectra showed the characteristic intense band at 1028 cm^−1^ with a shoulder at 1052 cm^−1^ and 986 cm^−1^ related to C-O-H and C-O-C stretching. Other identified signals were at 937 and 879 cm^−1^ (C-O-C stretching) and 1427 cm^−1^ (C-H bending of methylene groups). No relevant differences were observed between FT-IR spectra of inulin extracted by MAE and commercial inulin. Interestingly, the sharpness of the most intense band at 1028 cm^−1^ was higher in inulin from PER roots than in commercial roots. This can be likely related to a high purity of inulin extracted by MAE and the absence of other insoluble polymers co-extracted [9,51].

#### 3.3.3. Phenols and Inulin Productivity

The estimated phenols and inulin productivity (kg Ha^−1^) is reported in Table 3. These data were calculated by combining data of biomass availability in the field (Table 1) and extraction yields of phenols (Table 2) and inulin (Figure 3), respectively.

According to our data, the sequential microwave-assisted extraction of residual artichoke biomass left in the field (heads, leaves, stalks, and roots) would provide 176 kg Ha^−1^ of phenols and 4884 kg Ha^−1^ of inulin, overall. Therefore, the biorefinery applied to artichoke residues represents a relevant opportunity for valorizing an emerging agriculture waste in view of “closing the loop” approach postulated in the Circular Economy. These data are an estimation that cannot be taken as absolute values in view of the dependence of biomass productivity on many factors such as crop yield, space between plants, etc. However, they are useful to define the order of magnitude of the possible production of phenols and inulin referred to the field.

Moreover, phenols and inulin market continues to grow globally, making them attractive compounds in an economic point of view. The phenols market is expected to increase at an annual rate of 9% by 2020, and phenols consumption will increase from 12,200 tons to 33,880 tons by the end of 2025, corresponding to a market of EUR 1,760,000,000 [52].

On the other hand, the global inulin market size is likely to surpass EUR 1,690,000,000 by the end of 2025. Increasing demand for functional food ingredients to replace fat and sugar while improving mouthfeel and health is expected to increase inulin consumption in the food and beverage industry [53].

#### 3.3.4. Solid Residue Characterization for Potential Application as Bioenergy Feedstock

The sequential green extraction process of phenols and inulin from artichoke biomass also generated a final solid residue (IER) that amounted to 16.1 t Ha^−1^ (Table 3). According to Zuorro et al. [14], a possible valorization route could be bioenergy production through the combustion process. The use of biomass fuels provides substantial environmental benefits because it does not contribute to the greenhouse effect [54]. Table 4 reports the proximate and ultimate analysis of residues after the sequential extraction (IER).

IER of heads, leaves, and stalks showed chemical and physical characteristics comparable with data reported by Zuorro et al. [14] for artichoke residues, and other herbaceous feedstocks, such as wheat straw and corn stover, that are currently used for bioenergy production by combustion [55]. Moreover, the sequential extraction process resulted in an improvement of chemical characteristics of residual biomass as biofuel. Actually, a significant increase in carbon (C) and lignin content was found in IER of heads, leaves, and stalks (Table 4) compared to raw biomass (Table 1). This generated a consequent increase in HHV from 17.36 MJ kg^−1^ dw to 18.92 MJ kg^−1^ dw (average values), making IER attractive feedstocks for combustion. A critical aspect was represented by nitrogen (N) content, that was very high in heads and leaves (3.26 and 3.43% dw, respectively). This component is directly related to NOx emissions which lead to acid rain and ozone depletion. However, NOx emissions can be efficiently reduced by cofiring techniques using different biomass feedstocks [54].

On the other hand, IER of roots showed a very high content of ash (25.33% dw) and a low HHV (14.54 MJ kg^−1^ dw) that made it unusable for combustion.

#### 3.3.5. Solid Residue Characterization for Potential Application as Green Manure

Another possible route for valorizing the IERs (extracted with green solvents, then without environmental concerns) could be simply to return them back to the filed as green manure, improving soil fertility, and closing the loop in a circular way.

Soil management and farming strategies highly impact on soil fertility [56,57] and may affect ecological soil functions [58,59]. Furthermore, intensive management of agro-ecosystems has reduced the net amount of CO_2_ sequestered into the soil as soil organic carbon by increasing the mineralization rate of soil organic matter [60,61].

According to the European Green Deal, the achievement of sustainable soil management in the EU will be crucial for several of the planned actions. Some outlined practical actions for sustainable soil management include: arable land conversion to grassland, crop residues incorporation, reduced tillage, ley cropping, green manure and cover crops [62].

Therefore, returning extracted artichoke biomass to the field as green manure will perfectly fit with the EU actions, contributing to increasing organic matter and inorganic elements in the soil. Actually, the extracted residues (IERs) showed a high amount of organic matter (volatile solids, VS), carbon (C), and nitrogen (N) that ranged between 70.56 and 93.63% dw for VS, 35.08 and 45.37% dw for C, 1.23 and 3.43% dw for N, respectively (Table 5). Nevertheless, IERs showed also an interesting amount of macro- and microelements, including phosphorus (P), potassium (K), magnesium (Mg), and iron (Fe) (Table 5). Furthermore, no contamination by heavy metals was observed.

According to our data, if all IERs are returned to the field, about 13.77 t Ha^−1^ of organic matter will be supplied to the soil together with 6.74 t Ha^−1^ of carbon C, 352 kg Ha^−1^ of nitrogen N, 17.09 kg Ha^−1^ of phosphorous P and 117 kg Ha^−1^ of potassium K (Table 6). Concerning N supply to the soil, that is used as the key parameter for evaluating the effectiveness of cover crops, the N supply from IERs of artichoke biomass was higher than those reported for conventional cover crops including legume (28–238 kg Ha^−1^) and grass cover crops (23–348 kg Ha^−1^) [63]. Therefore, IERs could have great potential for sustainably improving soil fertility.

## 4. Conclusions

Annual cultivation of globe artichoke generates a considerable amount of waste (≈33 t Ha^−1^) that represents an intriguing source of biomass that can be converted into high-value compounds. Heads, leaves, stalks, and roots left in the field have been valorized through a biorefinery approach with sequential extractions, setting up an example of Circular Economy. A high amount of phenols (caffeoylquinic derivatives, flavones luteolin and apigenin) and inulin were sequentially extracted using green extraction techniques (MAE) and solvents making them directly usable for humans (nutraceutics, pharma, cosmetics) and animals (feed). Final extraction residues were effectively valorized through bioenergy production (combustion) and green manure (back to the field), improving soil fertility. The scalability of the entire process will be investigated in the upcoming research activities.

## Figures and Tables

**Table 1:** Availability of artichoke waste left in the field (Heads, Leaves, Stalks, and Roots), and proximate and ultimate analysis of raw biomass.

Plant Weight (Mean Value)	22.28 ± 4.32 (kg ww)				
Plant Components	Heads	Leaves	Stalks	Roots	Total Biomass
(% dw)	1.21 ± 0.09	32.17 ± 2.41	32.57 ± 2.44	34.04 ± 2.67	
t (ww) Ha^−1^	2.32 ± 0.14	80.27 ± 6.23	66.67 ± 5.62	32.65 ± 2.71	181.91 ± 13.64
t (dw) Ha^−1^	0.4 ± 0.03	10.55 ± 0.71	10.68 ± 0.83	11.16 ± 0.87	32.79 ± 2.63
Parameter					
Moisture (%)	82.81 ± 1.24	86.86 ± 1.52	83.98 ± 1.11	65.82 ± 0.87	
ASH (% dw)	7.63 ± 0.57	14.8 ± 0.62	6.4 ± 0.31	17.58 ± 0.76	
Volatile Solids (% dw)	75.39 ± 1.03	72.22 ± 0.93	74.99 ± 1.03	65.05 ± 0.87	
Fixed Carbon (% dw)	16.98 ± 0.86	12.98 ± 0.71	18.61 ± 0.92	17.37 ± 0.81	
C (% dw)	39.63 ± 0.26	40.23 ± 0.33	39.88 ± 0.29	36.25 ± 0.22	
H (% dw)	6.82 ± 0.13	6.31 ± 0.16	6.57 ± 0.12	6.18 ± 0.14	
N (% dw)	2.49 ± 0.16	2.77 ± 0.13	0.87 ± 0.09	0.74 ± 06	
S (% dw)	0.30 ± 0.08	0.43 ± 0.09	0.22 ± 0.03	0.18 ± 0.03	
O (% dw)	43.13 ± 0.27	35.46 ± 0.29	46.06 ± 0.32	39.07 ± 0.25	
Cl (% dw)	0.24 ± 0.01	0.25 ± 0.02	0.26 ± 0.02	0.31 ± 0.03	
HHV (MJ/kg dw)	17.43 ± 0.14	17.67 ± 0.12	16.98 ± 0.13	15.72 ± 0.15	
Protein (% dw)	15.58 ± 0.39	17.32 ± 0.43	5.44 ± 0.14	4.61 ± 0.18	
Total Carbohydrates (% dw)	56.42 ± 1.41	65.00 ± 1.66	60.86 ± 1.58	57.79 ± 1.32	
Cellulose (% dw)	18 ± 0.45	15.76 ± 0.39	24.15 ± 0.60	6.13 ± 0.15	
Hemicellulose (% dw)	8.27 ± 0.21	7.89 ± 0.26	10.86 ± 0.31	4.6 ± 0.15	
Lignin (% dw)	14.06 ± 0.39	10.78 ± 0.72	15.62 ± 0.91	13.52 ± 0.48	

**Table 2:** Chemical composition of phenolic extracts from different components of artichoke waste.

Compound	Heads	Leaves	Stalks	Roots
	*(mg/g dw)*	*(mg/g dw)*	*(mg/g dw)*	*(mg/g dw)*
*Chlorogenic Acid*	2.281 ± 0.114	3.891 ± 0.195	2.796 ± 0.140	1.403 ± 0.070
*Cynarin*	0.142 ± 0.007	0.036 ± 0.002	0.025 ± 0.001	0.156 ± 0.008
*Caffeic Acid*	0.178 ± 0.009	0.110 ± 0.006	0.108 ± 0.008	0.060 ± 0.003
*Luteolin 7-O-glucoside*	ND	0.608 ± 0.030	ND	ND
*Luteolin 7-O-glucoronide*	ND	0.545 ± 0.027	ND	ND
*Trans Ferulic Acid*	0.040 ± 0.002	0.177 ± 0.009	0.015 ± 0.004	0.504 ± 0.025
*1,5-di-O-Dicaffeoylquinic Acid*	2.783 ± 0.139	1.090 ± 0.055	2.450 ± 0.123	0.890 ± 0.045
*3,4-O Dicaffeoylquinic Acid*	0.805 ± 0.031	0.201 ± 0.026	0.516 ± 0.029	0.270 ± 0.025
*Luteolin 7-O-rutinoside*	ND	0.264 ± 0.019	ND	ND
*Apigenin 7-O-glucoside*	0.249 ± 0.037	0.016 ± 0.002	0.017 ± 0,004	ND
*Quercitin*	0.002 ± 0.001	0.006 ± 0.001	0.002 ± 0.001	0.002 ± 0.001
*Apigenin 7-O-glucoronide*	0.035 ± 0.003	0.002 ± 0.001	ND	ND
*Apigenin 7-O-rutinoside*	0.008 ± 0.001	ND	0.006 ± 0.001	ND
***Total Phenols***	**6.523 ± 0.326**	**6.943 ± 0.347**	**5.933 ± 0.297**	**3.283 ± 0.164**

ND: Not detected because below the instrumental detection limit.

**Table 3:** Phenols, inulin and residue productivity from artichoke waste biorefinery.

	Phenols	Inulin	Solid Residue (IER)
Plant components	kg Ha^−1^	kg Ha^−1^	t Ha^−1^
Heads	2.60 ± 0.09	26.52 ± 0.93	0.21 ± 0.01
Leaves	73.20 ± 2.56	158.22 ± 5.54	5.64 ± 0.19
Stalks	63.33 ± 2.22	96.12 ± 3.36	6.09 ± 0.21
Roots	36.61 ± 1.28	4602.72 ± 83.09	4.17 ± 0.15
Total Yield	**175.74 ± 6.15**	**4883.58 ± 91.13**	**16.10 ± 0.56**

**Table 4:** Proximate analysis of residual artichoke biomass after phenols and inulin extraction (IER), and comparison with other herbaceous feedstocks for bioenergy production.

	Heads	Leaves	Stalks	Roots	Artichoke Residues ^a^	Wheat Straw ^b,c^ (Range)	Corn Stover ^b,c^ (Range)
Moisture (%)	4.2	5.11	4.81	5.54	nr	2.55–7.36	2.46–8.07
ASH (% dw)	2.35	7.31	2.95	25.33	1.6	3.37–15.55	1.77–16.65
Volatile Solids (% dw)	88.29	87.8	93.63	70.56	nr	60.32–78.40	65.28–77.54
Fixed Carbon (% dw)	9.36	4.89	3.42	4.11	nr	9.14–23.84	10.80–22.97
C (% dw)	45.37	44.32	44.17	35.08	44.1	38.07–47.09	39.68–47.70
H (% dw)	6.52	6.52	6.33	5.13	6.3	4.05–6.58	4.31–8.68
N (% dw)	3.26	3.43	1.23	1.85	1.4	0.23–1.04	0.15–1.68
S (% dw)	0.12	0.22	0.05	0.06	<0.1	0.19–0.90	0.15–1.04
O (% dw)	42.38	38.19	45.27	32.55		37.52–47.35	36.13–49.17
Cl (% dw)	<0.01	<0.01	<0.01	<0.01	nr	0.15–1.50	0.15–1.20
Lignin (% dw)	28.62	27.99	21.85	40.46	nr	15.13–27.90	14.66–30.02
HHV (MJ kg^−1^ dw)	19.23	19.19	18.32	14.54	19.6	14.59–18.14	15.24–18.30
**Ash Fusibility**							
ST (Shrinkage, °C)	903	993	897	1003	nr	nr	nr
DT (Deformation, °C)	1360	1320	1365	1273	nr	950	1150
HT (Hemisphere, °C)	1390	1350	1390	1333	nr	nr	nr
FT (Flow, °C)	1480	1427	1463	1487	nr	1270	1280

^a^ Zuorro et al. 201. ^b^ Whang et al. 202. ^c^ Jha and Dass, 202. nr: not reported.

**Table 5:** Elemental composition of raw and residual biomass after phenolic and inulin extraction.

Element (mg/kg dw)	Raw Biomass				Residual Biomass			
	Heads	Leaves	Stalks	Roots	Heads	Leaves	Stalks	Roots
**Al**	33.31	200.37	52.26	618.28	216.37	797.67	139.02	1897.55
**As**	ND	ND	ND	0.46	ND	ND	ND	ND
**B**	14.46	22.65	14.98	8.71	13.91	16.07	11.26	10.80
**Ba**	ND	20.15	7.61	48.25	0.00	31.36	13.33	106.58
**Be**	ND	ND	ND	ND	ND	ND	ND	ND
**Ca**	2043.87	20,661.21	5700.07	3775.96	**3231.43**	**23,930.29**	**5656.15**	**6518.26**
**Cd**	ND	ND	ND	ND	ND	ND	ND	ND
**Co**	ND	ND	ND	ND	ND	ND	ND	ND
**Cr**	0.04	0.52	0.03	3.17	0.14	6.00	3.35	8.65
**Cu**	4.11	2.71	1.70	9.73	2.73	0.46	ND	16.50
**Fe**	72.34	425.84	393.59	2446.44	**74.81**	**603.48**	**246.45**	**3294.08**
**Hg**	ND	ND	ND	ND	ND	ND	ND	ND
**K**	13,717.60	30,739.82	17,866.30	5541.83	**9903.07**	**9402.07**	**7794.11**	**3526.07**
**Mg**	1765.90	1547.33	1055.82	278.36	**1630.21**	**704.38**	**534.77**	**426.21**
**Mn**	17.79	45.53	10.46	96.87	22.52	47.71	9.66	160.30
**Na**	13.52	4402.88	1209.16	811.20	115.68	1074.95	447.05	690.74
**Ni**	0.48	0.05	ND	1.34	ND	2.05	ND	2.74
**P**	3046.05	2017.00	1755.06	1811.49	**1459.67**	**1544.73**	**830.79**	**725.40**
**Pb**	ND	ND	ND	ND	ND	ND	ND	ND
**S**	1963.65	4843.28	1094.01	770.70	**1218.21**	**2242.07**	**485.56**	**647.14**
**Se**	ND	ND	ND	ND	0.41	0.51	0.48	0.24
**Si**	5386.15	5021.47	3181.10	10,076.69	**4963.29**	**12,607.87**	**4734.35**	**21,903.69**
**Sr**	1.71	63.56	24.58	32.92	3.67	76.61	30.80	68.91
**Ti**	2.87	31.35	4.79	228.91	2.39	60.42	7.05	389.03
**V**	ND	0.45	ND	8.86	ND	0.38	ND	13.39
**Zn**	5.47	ND	ND	ND	ND	ND	ND	ND

ND: Not detected because below the instrumental detection limit.

**Table 6:** Organic matter and carbon (C), nitrogen (N), phosphorus (P) and potassium (K) supply from artichoke plant residues returned to the soil after sequential extraction of phenols and inulin.

	Organic Matter	C	N	P	K
	t Ha^−1^	t Ha^−1^	kg Ha^−1^	kg Ha^−1^	kg Ha^−1^
Heads	0.19 ± 0.01	0.10 ±0.01	6.84 ± 0.24	0.31 ± 0.02	2.08 ± 0.07
Leaves	4.95 ± 0.17	2.50 ± 0.09	193.29 ± 6.77	8.71 ± 0.30	52.98 ± 1.85
Stalks	5.70 ± 0.20	2.69 ± 0.09	74.91 ± 2.62	5.06 ± 0.18	47.46 ± 1.66
Roots	2.94 ± 0.10	1.46 ± 0.05	77.07 ± 2.70	3.02 ± 0.11	14.69 ± 0.51
Total	13.77 ± 0.48	6.75 ± 0.24	352.11 ± 11.32	17.09 ± 0.60	117.22 ± 4.10

## Data Availability

Data is contained within the article or Supplementary Materials.

## Supplementary Materials

The following are available online at https://www.mdpi.com/2304-8158/10/1/112/s1, Figure S1: Total phenols yields (mg g^−1^ dw) achieved by MAE at selected best conditions (EtOH 25% *v/v* in water; 5 min; 50 °C); Figure S2: Infrared spectra (FT-IR ATR) of inulin extracted by MAE from raw roots (purple line) and PER of roots (yellow line) compared with commercial inulin (Sigma–Aldrich) from chicory (grey line).
