# Supplementary material for: Artichoke Biorefinery: From Food to Advanced Technological Applications
Source: Foods. 2021 Jan 7;10(1):112. doi: 10.3390/foods10010112 (PMC7827807; doi:10.3390/foods10010112)
Supplement: Supplementary file 1 [file foods-10-00112-s001.pdf]

Figures Supplemetary Material

Figure S1

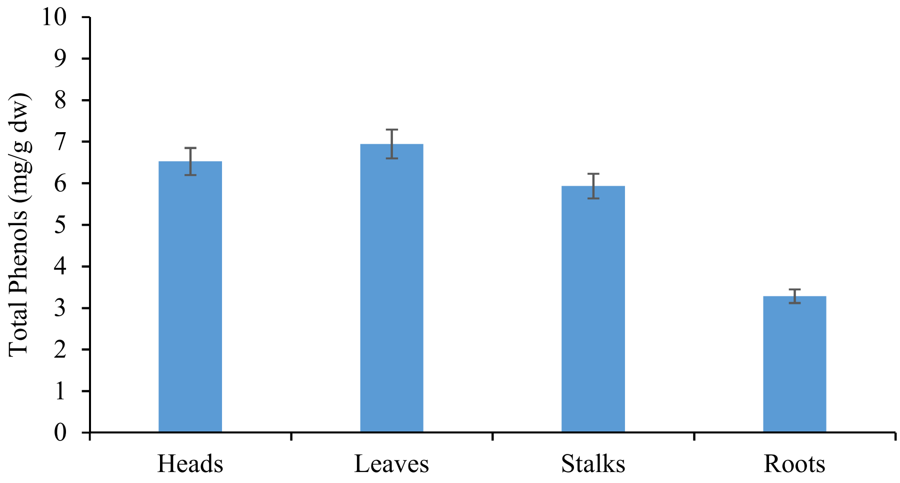

Figure S1. Total phenols yields (mg g^{-1} dw) achieved by MAE at selected best conditions (EtOH 25% V/V in water; 5 minutes; 50 °C)

Figure S2.

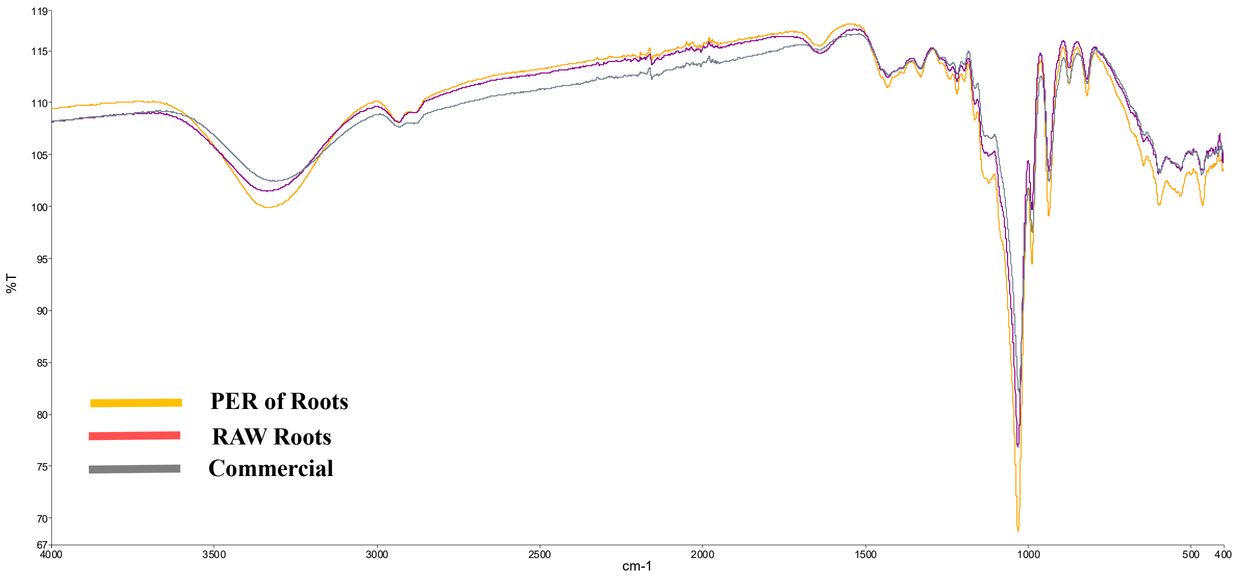

Figure S2. Infrared spectra (FT-IR ATR) of inulin extracted by MAE from raw roots (purple line) and PER of roots (yellow line) compared with commercial inulin (Sigma Aldrich) from chicory (grey line).

Tables Supplementary Material

Table S1. Calibration curves and calculation of limit of detection (LOD) and limit of quantification (LOQ) values.

Compound	Calibration Range (5 levels)	Slope of Calibration curve	Standard Deviation σ	Limit of detection (LOD) = 3.3σ/S	Limit of quantitation (LOQ) = 10σ/S
	(µg/mL)			*(µg/mL)*	*(µg/mL)*
Chlorogenic Acid	5-250	41670.0516	56.5587	0.0041	0.0136
Cynarin	5-250	45865.2479	38.3039	0.0025	0.0084
Caffeic Acid	5-250	102815.8158	213.9416	0.0062	0.0208
Luteolin 7-O-glucoside	0.5-25	95473.7190	113.0524	0.0036	0.0118
Luteolin 7-O-glucoronide	0.5-25	98561.7391	103.0524	0.0031	0.0105
Trans Ferulic Acid	5-250	13879.2399	55.4489	0.0120	0.0400
1,5-di-O-Dicaffeoylquinic Acid	5-250	45864.3967	38.6466	0.0025	0.0084
3,4-O Dicaffeoylquinic Acid	5-250	45863.4711	38.5804	0.0025	0.0084
Luteolin 7-O-rutinoside	0.5-25	95451.7419	73.0524	0.0023	0.0077
Apigenin 7-O-glucoside	0.5-25	95471.5868	112.9977	0.0036	0.0118
Quercitin	5-250	184676.3168	171.9757	0.0028	0.0093
Apigenin 7-O-glucoronide	0.5-25	97369.5828	110.0362	0.0034	0.0113
Apigenin 7-O-rutinoside	0.5-25	95593.6194	108.0571	0.0034	0.0113

Table S2. Database of Total Phenols yields achieved from artichoke plant residues (Heads, Leaves, Stalks and Roots) at different experimental extraction conditions.

	Time	Extraction condition	Water		EtOH 25%		EtOH 50%		EtOH 75%	
			TP mean value (mg/g dw)	StDev	TP mean value (mg/g dw)	StDev	TP mean value (mg/g dw)	StDev	TP mean value (mg/g dw)	StDev
Heads	20'	25°C	2.003	0.200	3.529	0.353	4.800	0.480	4.088	0.409
Heads	8h	25°C	0.306	0.018	2.483	0.149	4.944	0.297	4.261	0.256
Heads	24h	25°C	0.236	0.017	1.168	0.082	4.770	0.334	4.088	0.286
Heads	48h	25°C	0.145	0.007	0.570	0.029	4.481	0.224	3.421	0.171
Heads	5'	50°C Mw	5.434	0.272	6.523	0.326	6.659	0.333	6.830	0.341
Heads	10'	50°C Mw	4.446	0.311	6.665	0.467	6.771	0.474	6.021	0.421
Heads	20'	50°C Mw	4.782	0.383	6.768	0.541	6.906	0.552	7.061	0.565
Heads	5'	75°C Mw	3.653	0.219	6.038	0.362	6.209	0.373	5.514	0.331
Heads	10'	75°C Mw	3.158	0.316	5.801	0.580	6.485	0.649	5.624	0.562
Heads	20'	75°C Mw	2.589	0.129	5.500	0.275	5.918	0.296	5.362	0.268
Heads	5'	100°C Mw	2.993	0.120	6.231	0.249	7.157	0.286	5.949	0.238
Heads	10'	100°C Mw	4.095	0.287	6.779	0.475	7.202	0.504	6.401	0.448
Heads	20'	100°C Mw	3.499	0.175	6.436	0.322	6.860	0.343	5.806	0.290
Leaves	20'	25°C	0.695	0.069	3.903	0.390	4.349	0.435	4.210	0.421
Leaves	8h	25°C	0.297	0.018	2.125	0.127	4.559	0.274	3.671	0.220
Leaves	24h	25°C	0.184	0.013	1.180	0.083	4.371	0.306	3.885	0.272
Leaves	48h	25°C	0.279	0.014	0.779	0.039	4.003	0.200	3.676	0.184
Leaves	5'	50°C Mw	4.291	0.215	6.943	0.347	6.332	0.317	4.997	0.250
Leaves	10'	50°C Mw	3.814	0.267	6.380	0.447	6.536	0.457	5.636	0.395
Leaves	20'	50°C Mw	3.534	0.283	6.555	0.524	7.187	0.575	6.346	0.508
Leaves	5'	75°C Mw	3.194	0.192	7.028	0.422	6.085	0.365	5.232	0.314
Leaves	10'	75°C Mw	2.956	0.296	7.124	0.712	6.739	0.674	5.919	0.592
Leaves	20'	75°C Mw	3.103	0.155	6.665	0.333	6.355	0.318	5.876	0.294
Leaves	5'	100°C Mw	3.650	0.146	6.559	0.262	7.144	0.286	5.939	0.238
Leaves	10'	100°C Mw	4.184	0.293	6.658	0.466	7.283	0.510	6.328	0.443

Leaves	20'	100°C Mw	3.789	0.189	6.135	0.307	7.033	0.352	5.704	0.285
Stalks	20'	25°C	3.191	0.319	3.838	0.384	4.185	0.418	3.599	0.360
Stalks	8h	25°C	2.018	0.121	3.478	0.209	4.333	0.260	3.413	0.205
Stalks	24h	25°C	1.238	0.087	3.692	0.258	4.231	0.296	3.184	0.223
Stalks	48h	25°C	0.796	0.040	3.660	0.183	4.111	0.206	3.337	0.167
Stalks	5'	50°C Mw	2.621	0.131	5.933	0.297	5.815	0.291	5.457	0.273
Stalks	10'	50°C Mw	3.034	0.212	5.884	0.412	5.435	0.380	5.897	0.413
Stalks	20'	50°C Mw	3.393	0.271	5.792	0.463	5.354	0.428	5.287	0.423
Stalks	5'	75°C Mw	2.342	0.141	6.368	0.382	5.096	0.306	5.521	0.331
Stalks	10'	75°C Mw	3.071	0.307	6.792	0.679	5.930	0.593	5.646	0.565
Stalks	20'	75°C Mw	3.275	0.164	5.904	0.295	6.914	0.346	6.050	0.302
Stalks	5'	100°C Mw	2.629	0.105	6.779	0.271	6.084	0.243	6.255	0.250
Stalks	10'	100°C Mw	4.435	0.310	6.732	0.471	7.150	0.500	7.038	0.493
Stalks	20'	100°C Mw	3.886	0.194	4.518	0.226	6.363	0.318	6.259	0.313
Roots	20'	25°C	0.623	0.062	1.004	0.100	1.110	0.111	0.793	0.079
Roots	8h	25°C	0.097	0.006	0.427	0.026	1.670	0.100	1.645	0.099
Roots	24h	25°C	0.026	0.002	0.060	0.004	1.307	0.091	1.871	0.131
Roots	48h	25°C	0.018	0.001	0.034	0.002	0.927	0.046	1.560	0.078
Roots	5'	50°C Mw	1.344	0.067	3.283	0.164	2.660	0.133	3.031	0.152
Roots	10'	50°C Mw	1.752	0.123	2.470	0.173	2.918	0.204	2.818	0.197
Roots	20'	50°C Mw	1.022	0.082	2.871	0.230	3.036	0.243	2.322	0.186
Roots	5'	75°C Mw	2.015	0.121	3.199	0.192	2.765	0.166	2.321	0.139
Roots	10'	75°C Mw	1.949	0.195	2.984	0.298	3.117	0.312	2.705	0.270
Roots	20'	75°C Mw	2.375	0.119	2.821	0.141	2.994	0.150	3.395	0.170
Roots	5'	100°C Mw	1.127	0.045	3.011	0.120	3.590	0.144	2.493	0.100
Roots	10'	100°C Mw	2.100	0.147	3.712	0.260	4.391	0.307	3.717	0.260
Roots	20'	100°C Mw	2.333	0.117	3.526	0.176	3.335	0.167	3.638	0.182
